# On an Effectual Antidote to Strychnia

**Published:** 1849-01

**Authors:** Isaac Pidduck

**Affiliations:** London


*On an Effectual Antidote to Strychnia.* By Isaac Pidduck, M. D., London.—As an antidote to the poison of strychnia, camphor is effectual. The fourth part of a grain of strychnia (instead of the sixteenth, which had been prescribed for neuralgic pains) was taken by a weakly man. His muscles were convulsed with tetanic spasms. Five grains of camphor were dissolved in almond emulsion, and almost immediately after taking this dose the spasms ceased.

I am induced to send this communication in consequence of the fatal event which recently occurred near Romsey, and also of a distressing case at Edinburgh, in which the sufferings of the patient did not terminate until the influence of the poison was exhausted. In the former case, there was no opportunity for the administration of an antidote, for the patient was found dead by Mr. Taylor, the surgeon of Romsey; but in the latter case the patient might have been more speedily relieved from the effects of an over-dose of strychnia. I may add that Dr. Anderson's case tends to confirm my statement of the efficacy of strychnia in the cure of certain forms of neuralgia, some cases of which I published in the Medical Gazette in 1840.—*London Lancet.*

